# Memory and Learning Complaints in Relation to Depression among Elderly People with Multimorbidity

**DOI:** 10.3390/geriatrics2020015

**Published:** 2017-05-09

**Authors:** Bishwajit Ghose, Mahaman Yacoubou Abdoul Razak

**Affiliations:** 1 School of Medicine and Health Management, Tongji Medical College, Huazhong University of Science and Technology, Wuhan 430030, China; 2 Department of Pathophysiology, School of Basic Medicine and the Collaborative Innovation Center for Brain Science, Key Laboratory of Ministry of Education of China for Neurological Disorders, Tongji Medical College, Huazhong University of Science and Technology, Wuhan 430030, China; razakmahaman@yahoo.fr

**Keywords:** memory and learning complaints, depression, elderly people, multimorbidity, South Africa

## Abstract

Although current models of care are generally well-suited to providing treatment for individual medical conditions, the emergence of multimorbidity is becoming a serious concern for practitioners and policy researchers, particularly in developing countries. The challenges of tackling multimorbidity are further compounded when the multimorbidity co-occurs with psychiatric conditions such as cognitive and depressive disorders. Understanding the relationships between multimorbidity and psychiatric illnesses is therefore of considerable clinical importance. In the present study, we cross-sectionally examined whether multimorbidity has an association with perceived cognition—including memory, learning complaints, and depression—among elderly population in South Africa. Study subjects were 422 men and women aged 50 years and older. The prevalence of arthritis, asthma, cancer, diabetes, heart disease, chronic lung disease, hypertension, and stroke was respectively 31.5, 7.3, 1.7, 10.2, 1.2, 1.7, 52.1, and 31.5%, and that of multimorbidity was 30.8%. In the multivariate analysis, women with multimorbidity were 4.33 times (OR = 4.33, 95%CI = 2.96–14.633) more likely to report memory complaints. The odds of diagnosed depression were 1.4 times (OR = 1.4, 95%CI = 1.045–5.676), and the odds of self-reported depression were 1.7 times (OR = 1.7, 95%CI = 1.41–2.192) higher among women who had multimorbidity compared with those who had no morbid conditions. However, the association was not significant among men. Overall, the findings suggest that the occurrence of multimorbidity warrants special attention, especially regarding its compounding effects on psychological health. The findings need to be replicated through longitudinal studies that consider a broader range of chronic conditions.

## 1. Introduction

Multimorbidity, in general terms referring to the coexistence of more than one chronic condition in the same person, is becoming a major concern for healthcare systems, especially in resource-poor countries. This is largely because primary care settings are typically configured to providing services for individual disease conditions rather than multiple diseases presenting in the same patient [1]. As a population experiences longer life spans, attributable to the gradually reducing burden of mortality from acute infectious diseases, older people are more likely to develop at least one or more chronic conditions with subsequent impacts on subjective health, reduced quality of life, higher rate of medication use, and hospital admissions [2,3,4,5] . Reduced physical capacity and increased dependency can further compromise an individual’s social, emotional, and cognitive health, and thereby affect his or her psychological well-being. A growing body of research supports the association of ageing with a higher prevalence of psychological disorders [6,7], of which cognitive decline and Major Depressive Disorder (MDD) are among the most frequent [8,9].

MDD and mild or brief depressive episodes are regularly encountered in primary care settings among the elderly, and are considered a global health issue. However, definitive evidence on the causal relationship between ageing and depression is still lacking. Less controversially, old age can exert negative effects on mental health outcomes through the occurrence of physical morbidity/co-morbidity and associated disabilities. However, the current literature regarding the relationship between multimorbidity and mental illness is inconclusive, particularly in countries in Sub-Saharan Africa. For this reason, an attempt was made in the present study to examine whether or not occurrences of single or multiple morbidity are associated with depression in the elderly population in South Africa. Because of the scarcity of relevant primary data, we used secondary data from the South Africa—SAGE Well-Being of Older People Study (WOPS) that had been conducted in 2010.

## 2. Methods

### 2.1. Data Source

This study was based on secondary data obtained from South Africa—SAGE Well-Being of Older People Study-2010 (WOPS) Wave 1. The WOPS cross-sectional survey was designed by the World Health Organization and the Africa Centre for Health and Population Studies and implemented by the Africa Centre for Health and Population Studies [10]. The main objectives of the survey were to provide evidence on self-reported physical and mental health status along with other sociodemographic and disease conditions among elderly people infected and/or affected by HIV. The sample population comprised 422 men and women aged 50 years and above residing within the Africa Centre surveillance area. Subjects were interviewed face to face between March–August 2010. Details of sampling area, procedures, and study objectives have been published elsewhere [11].

### 2.2. Variables

The principal outcome variables were classified into two sub-domains, including cognitive complaints and depression, each of which were evaluated by two self-reported questions:

1. Difficulty experienced last 30 days, concentrating/remembering things
2. Difficulty experienced last 30 days, learning a new task

And,

1. Ever diagnosed with depression
2. Felt sad, empty, or depressed last 12 months

To assess cognitive complaints, participants were asked: (1) “Overall, in the last 30 days, how much difficulty did you have with concentrating or remembering things? (e.g. cooking, bathing);” and (2) “Overall, in the last 30 days, how much difficulty did you have in learning a new task (for example, learning how to get to a new place)?” Subjects were given the following choices of response: none/mild/moderate/severe/extreme/cannot do.

To assess depression, participants were asked: (1) “Have you ever been diagnosed with depression?” and (2) “During the last 12 months, have you had a period lasting several days when you felt sad, empty, or depressed?” Here subjects were asked to respond ‘yes’ or ‘no’.

The principal explanatory variable was the frequency of the occurrence (self-reported) of following chronic diseases: arthritis, asthma, cancer, diabetes, heart disease, chronic lung disease, hypertension, stroke. Frequencies were categorized as: no morbidity (0 conditions), single morbidity (1 condition), and multimorbidity (>1 condition).

To account for possible confounding effects, the following covariates were considered: age (50–59, 60–69, 70–79, 79+), gender (female, male), marital status (married, divorced/widowed, never married), educational attainment (nil, adult education only, up to grade 12), financial situation compared to 3 years ago (better, about the same, much worse), current tobacco use (daily, not daily, none), average times had an alcoholic drink during last 12 months (<1/month, 1– 7 days/month, 1–4 days+/week, none), Body Mass Index (eutrophic, overweight, obese), self-reported health (very good, good, moderate, bad, very bad).

### 2.3. Analytical Procedure

Basic characteristics of the study population were presented as crude percentages. All estimations were stratified by gender. A contingency table was generated to facilitate the comparison of the prevalence rates of cognitive complaints and depression across sociodemographic variables. Chi-square bivariate tests of association were used to calculate the p-values. Frequency of diseases was trichotomised as ‘none’, ‘1’, and ‘>1’. The categories of memory and learning complaints were collapsed into- ‘none’, ‘mild/moderate’, and ‘severe/extreme’. Multivariate regression models were then used to calculate the odds ratios of the association (from adjusted models) between the outcome variables with the frequency of diseases by treating those with no diseases as the reference category. A two-tailed p-value of <0.05 was set as level of significance for all calculations. All analyses were carried out using R statistical software.

### 2.4. Ethics Statement

The WOPS survey was approved by the Research Ethics Committee of the University of KwaZulu-Natal. Informed written consent was obtained from all WOPS participants prior to the interviews.

## 3. Results

### 3.1. Prevalence Rates

The prevalence of arthritis, asthma, cancer, diabetes, heart disease, chronic lung disease, hypertension, and stroke was respectively 31.5, 7.3, 1.7, 10.2, 1.2, 1.7, 52.1, and 31.5%. Regarding cognitive problems, prevalences of mild/moderate and severe/extreme memory complaints were respectively 80.6% and 8.3%, and prevalences of learning complaints were 60.7% and 2.8% respectively. Diagnosed depression was reported by 2.8%, and perceived depression (during last 12 months) was reported by 51.9% of the participants.

**Figure 1:**
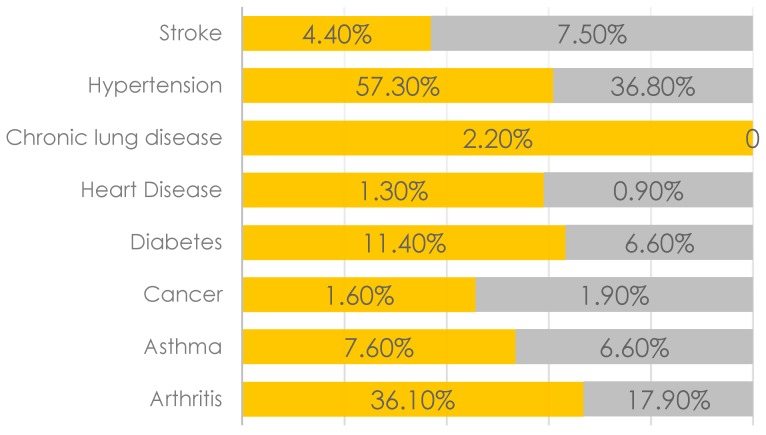
Prevalence rates of NCDs among women (in orange) and men (in grey).

**Figure 2:**
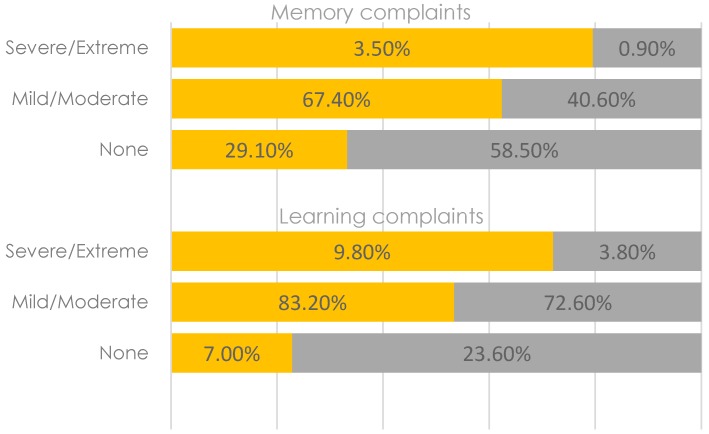
Prevalence rates of memory and learning complaints among women (in orange) and men (in grey).

**Figure 3:**
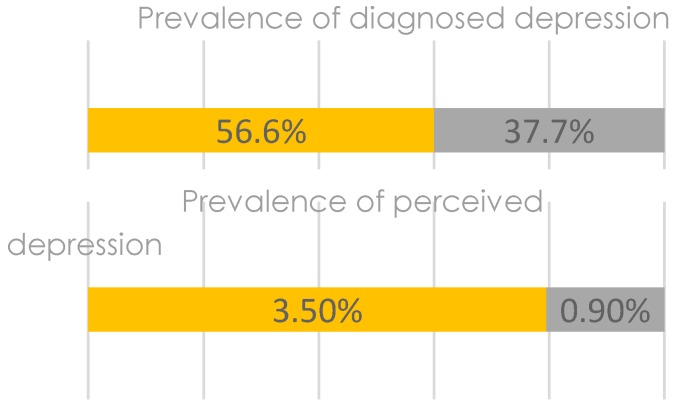
Prevalence rates of depression among women (in orange) and men (in grey).

Figure 1 illustrates that majority of the diseases were more prevalent among women, except for cancer (1.6% vs. 1.9%) and stroke (4.4% vs. 7.5%). However, the difference was statistically significant for hypertension only (result not shown). Similar patterns were observed for cognitive complaints (Figure 2) and depression as well (Figure 3).

Compared to men, women had higher rates of memory complaints (mild/moderate 83.2% vs. 72.6%, and severe/extreme 9.8% vs. 3.8%), learning complaints (mild/moderate 67.4% vs. 40.6% and severe/Eextreme 3.5% vs. 0.9%), diagnosed depression (3.5% vs. 0.9%) and self-reported depression (3.5% vs. 0.9%). No significant differences were observed in the prevalence rates of depression or memory and learning complaints.

### 3.2. Participant Characteristics

Table 1 indicates that most of the participants were 50–59 years of age (45.7%). Of the 422 participants, about three-quarters were female (74.9%). About half were widowed/divorced, and more than a quarter were currently married (27.5%). More than two-fifths had no formal education (41.2%), and more than half had Grade-12-level qualification. The overall rate of literacy was higher among men compared to women (44.6% vs. 31.1%). 51.9% of the participants reported experiencing worse economic conditions compared to three years previously. The prevalence of daily smoking was 7.8%, and that of occasional smoking was 3.6%. 86.5% of the sample reported never drinking, and a similar percentage, 88.6%, reported never smoking. Regarding body weight status, only 10.2% of the participants had a BMI value within healthy range (18.5–24.9 kg/m^2^), while about two-thirds (64%) were obese. The majority of participants rated their health status as neither good nor bad, with 1.4% reporting very good health and 1.2% very bad health. Men were more likely to rate their health status as very good/good compared to women (35.9% vs 18.4%). The prevalences of single and multimorbidity were 40.5% and 30.8%, respectively.

### 3.3. Association between Mulmorbidity with Cognitive Problem and Depression

Results of the multivariate regression analyses on the association between multimorbidity with cognitive problems and depression are presented in Table 2 and Table 3. The associations were significant for women only. Table 2 shows that women with multimorbidity were 4.33 times [OR = 4.33, 95%CI = 2.96–14.633] more likely to report memory complaints. The odds of having diagnosed depression were 1.4 times higher among women who had multimorbidity compared to those who had no morbid conditions (OR = 1.4, 95%CI = 1.045–5.676); the corresponding odds for self-reported depression were 1.7 times (OR = 1.7, 95%CI = 1.41–2.192) higher among women who had multimorbidity compared with those who had no morbid conditions. No statistically significant association was found between multimorbidity with cognitive disorder and depression among men.

## 4. Discussion

Using data from the South Africa—SAGE Well-Being of Older People Study (WOPS 2010)—the present study investigated the association between multimorbidity with cognitive problems and depression in a sample of the elderly population in South Africa. Because the data were cross-sectional and not representative of the general population, no causal inference can be drawn regarding the associations. However, the analyses did reveal several important findings. First, multimorbidity appeared to be a relatively common condition, as about thirty percent of the men and women were found to be living with more than one chronic disease. The prevalence of hypertension, more than 50%, was notably high.

Apart from hypertension, nearly one-third of the participants had experienced arthritis and stroke. Taken together, these figures reflect a high burden of Non-Communicable Diseases (NCDs) in the sample population. Since we included only eight types of chronic illnesses, the actual prevalence of NCDs might be even higher if a wider range of conditions were to be considered [12]. For example, the South Africa National Income Dynamic Survey (SA-NIDS) comprising five NCDs (tuberculosis, high blood pressure, diabetes, stroke, asthma, and cancer) reported a prevalence of multimorbidity of 4% with diabetes being the most and hypertension being the least prevalent [5]. A more recent study included a broader range of NCDs, and reported that the prevalence was 14%, with hypertension being the most frequent morbidity [13]. However, those findings are not comparable with the present study as we included elderly subjects only.

Of note, the prevalences of most of the chronic diseases were higher among women; women also had more than twice the prevalence of multimorbidity compared with their male counterparts. Similar results regarding gender disparity in chronic disease have been observed in a large number of studies using both younger and elderly subjects [13,14,15]. These findings suggest gender differences in vulnerability or rates of exposure to the risk factors, as well as differences in access to or utilization of preventive measures. Hence, special priority needs to be given to addressing gender-related factors in the design of future prevention and intervention programs.

Apart from the NCDs, the rates of cognitive disorder and depression also appeared to be higher among women. This finding might reflect the higher rate NCDs among women as revealed in the multivariable analyses of this study. Women with multimorbid conditions had significantly higher odds of having memory complaints, being diagnosed with depression, and of having perceived depression during the preceding 12 months. Moreover, in the regression analysis, it was observed that the association between cognitive disorder and depression with multimorbidity was significant only among women; the association between cognitive disorder and depression was significant only with single morbidity among men. This variation is challenging to explain within the scope of the present study. However, a possible contributing factor might be the tendency of women to report poorer self-rated physical and mental health than men [16,17].

The interrelation between multimorbidity and physiological health has been assessed by previous researchers in higher-income countries. In Australia, a primary-care cohort study found that the prevalence of depression increased with the number of NCDs, concluding that a dose-response relationship existed between the number of NCDs and depressive symptoms [18]. Similar findings were reported by studies in Singapore [19], the Netherlands [20], Germany [21], and the UK [22]. A case-control investigation on a representative Scottish population studied 32 common chronic conditions, reporting that depression was significantly associated with multimorbidity, adjusting for age, sex, and deprivation [23]. It is important to note here that the directionality of the association between depression and NCDs cannot correctly be determined from cross-sectional surveys, since outcome and exposure status are measured at the same time. It is also possible that the reverse affect can obtain.

The findings of our study contribute to current knowledge and have potential implications for the creation of new health policy in South Africa. However, there are several limitations. As mentioned earlier, the survey was cross-sectional and therefore cannot find causality between the outcome and exposure variables. The prevalence rates are not generalizable for the elderly population in the country. The variables were self-reported and thereby subject to reporting bias. Moreover, we could not adjust the analysis for multiple medication use (polypharmacy), a circumstance that is common among patients with multimorbidity and is itself associated with adverse physical and mental health outcomes. To date, only a small number of countries have been able to conduct investigations in this area, perhaps owing to the inherent complexity of the task. To our knowledge, there is no publicly available dataset on antipsychotic polypharmacy from any country.

## 5. Conclusions

In conclusion, the prevalence rates of multimorbidity, cognitive disorder, and perceived depression were high among the participants. Women had higher burdens for most of the chronic conditions, including memory and learning complaints and perceived depression. Finally, multivariate analysis indicated a strongly positive association between multimorbidity with memory complaints and perceived depression among women. Conclusions from the present study need to be verified by longitudinal studies that include larger samples and a wider range of chronic conditions. However, our findings do suggest that the elderly population in South Africa should be the focus of research and policy attention to explain and address the high burden of NCDs and their aggravating effects on mental health status, especially among women.

## Figures and Tables

**Table 1:** Sample characteristics, South Africa-WOPS 2010.

Variables	N (%)	Female (74.9%)	Male (25.1%)	*p*
**Age** (62.27 ± 10.09)		(62.44 ± 10.23)	(61.79 ± 9.70)	0.935
50–59	193 (45.7)	44.9	48.1	
60–69	129 (30.6)	30.7	30.2	
70–79	70 (16.6)	17.1	15.1	
79+	30 (7.1)	7.3	6.6	
**Marital status**				0.18
Married	116 (27.5)	28.2	25.5	
Divorced/widowed	210 (49.8)	46.2	60.4	
Never married	96 (22.7)	25.6	14.2	
**Educational attainment**				0.018
Nil	174 (41.2)	44.6	31.1	
Adult education only	27 (6.4)	5.1	10.4	
Up to grade 12	221 (52.4)	50.3	58.5	
**Financial situation compared to 3 Years ago**				0.89
Better	67 (15.9)	15.8	16.0	
About the same	136 (32.2)	31.6	34.0	
Much worse	219 (51.9)	52.5	50.0	
**Current tobacco use**				<0.0001
Daily	33 (7.8)	6.0	13.2	
Not daily	15 (3.6)	1.9	8.5	
No	374(88.6)	92.1	78.3	
Average times had alcoholic drink last 12 months *				<0.0001
<1 a month	23 (5.5)	2.5	14.2	
1– 7 days/month	21 (5.0)	3.5	9.4	
1–4 days/week	13 (3.1)	1.9	6.6	
None	365 (86.5)	92.1	69.8	
**BMI**				<0.0001
Normal weight	43 (10.2)	8.9	14.2	
Overweight	109 (25.8)	21.2	39.6	
Obese	270 (64.0)	69.9	46.2	
**SRH**				<0.0001
Very good	6 (1.4)	0	5.7	
Good	90 (21.3)	18.4	30.2	
Moderate	232 (55.0)	56.3	50.9	
Bad	88 (20.9)	23.4	13.2	
Very bad	5 (1.2)	1.6	0	
**Frequency of NCDs**				<0.0001
0	121 (28.7)	25.0	39.6	
1	171 (40.5)	39.2	44.3	
>1	130 (30.8)	35.8	16.0	

N.B. * = Standard size. SRH = Health in last two weeks. SRH = Self-rated health. NCDs = Non-communicable chronic diseases.

**Table 2:** Multivariate association between multimorbidity with memory and learning complaints among elderly men and women in South Africa, WOPS 2010.

No. of morbidities	Memory complaints		Learning complaints	
	Women (OR, 95%CI)	Men (OR, 95%CI)	Women (OR, 95%CI)	Men (OR, 95%CI)
None	Ref	Ref	Ref	Ref
One	9.37 (0.423–14.428)	2.667 * (1.392–4.021)	2.667 (0.337–21.116)	3.136 * (1.003–9.807)
Multiple	4.33 * (2.96–14.633)	1.131 (0.324–3.952)	1.683 (0.683–6.683)	1.752 (0.773–3.970)

N.B. * = results are statistically significant. Models adjusted for age, marital status, educational attainment, financial situation compared to 3 years ago, current tobacco use, average times had alcoholic drink last 12 months, BMI, and SRH.

**Table 3:** Multivariate association between multimorbidity with diagnosed and perceived depression, WOPS 2010.

No. of morbidities	Depression diagnosis		Perceived depression	
	Women (OR, 95%CI)	Men (OR, 95%CI)	Women (OR, 95%CI)	Men (OR, 95%CI)
None	Ref	Ref	Ref	Ref
One	0.967 (0.532–1.756)	1.849 * (1.515–3.40)	0.867 (0.470–1.602)	1.332 (0.148–2.971)
Multiple	1.400 * (1.045–5.676)	1.032 (0.307–3.470)	1.700 * (1.41–2.192)	0.906 (0.146–3.612)

N.B. * = results are statistically significant. Models adjusted for age, marital status, educational attainment, financial situation compared to 3 years ago, current tobacco use, average times had alcoholic drink last 12 months, BMI, and SRH.
